# CB2 and GPR55 Receptors as Therapeutic Targets for Systemic Immune Dysregulation

**DOI:** 10.3389/fphar.2016.00264

**Published:** 2016-08-22

**Authors:** Juan Zhou, Ian Burkovskiy, Hyewon Yang, Joel Sardinha, Christian Lehmann

**Affiliations:** ^1^Department of Anesthesia, Dalhousie UniversityHalifax, NS, Canada; ^2^Department of Microbiology and Immunology, Dalhousie UniversityHalifax, NS, Canada; ^3^Department of Pharmacology, Dalhousie UniversityHalifax, NS, Canada

**Keywords:** endocannabinoid system, immune dysfunction, sepsis, central nervous system injury, immunosuppression, GPR55, CNS injury-induced immunodeficiency syndrome

## Abstract

The endocannabinoid system (ECS) is involved in many physiological processes and has been suggested to play a critical role in the immune response and the central nervous system (CNS). Therefore, ECS modulation has potential therapeutic effects on immune dysfunctional disorders, such as sepsis and CNS injury-induced immunodeficiency syndrome (CIDS). In sepsis, excessive release of pro- and anti-inflammatory mediators results in multi-organ dysfunction, failure, and death. In CIDS, an acute CNS injury dysregulates a normally well-balanced interplay between CNS and the immune system, leading to increased patients’ susceptibility to infections. In this review, we will discuss potential therapeutic modulation of the immune response in sepsis and CNS injury by manipulation of the ECS representing a novel target for immunotherapy.

## Introduction

The endocannabinoid system (ECS) is involved in many physiological processes including metabolism, inflammation, pain, and neurotransmission (De Petrocellis and Di Marzo, 2009; Pandey et al., 2009). It consists of endogenous cannabinoids (EC), cannabinoid receptors (CBR), and EC metabolizing enzymes (De Petrocellis and Di Marzo, 2009; Pertwee et al., 2010). Two major CBRs are identified: CBR type 1 (CB_1_R) and CBR type 2 (CB_2_R; Howlett et al., 2002). CB_1_R are mainly expressed in the central nervous system (CNS) and a variety of non-neural peripheral tissues, including the vasculature and gut (Pertwee and Ross, 2002). CB_2_R are primarily expressed on immune cells but are also identified in selected CNS areas and some peripheral tissues (Klein, 2005). Recently another G protein-coupled receptor, GPR55, has garnered much attention due to its activation by EC and its impact on the immune system (Pertwee, 2007; Yang et al., 2016a). Increasing evidence shows that enhanced EC levels occur during systemic inflammation, such as in sepsis or following a CNS injury. Manipulation of the ECS may have therapeutic effects in inflammatory disorders. In this review, we will focus on modulation of CB_2_ and GPR55 receptors on immune response in two inflammatory disorders, sepsis, and CNS injury. Both sepsis and CNS injury cause the immune system to go through rather rapid and dramatic changes from pro- to anti-inflammatory phases, which may end up costing patients their life. We suggest that the common mechanism for modulating and ultimately controlling the response of the immune system can be achieved through delicate interplay between the endocannabinoid, central nervous and immune systems.

## ECS in Sepsis

Sepsis is defined as a life-threatening organ dysfunction caused by a dysregulated host response to an infection (Singer et al., 2016). An initial infection with the ensuing systemic inflammatory response becomes amplified, resulting in excessive release of both pro- and anti-inflammatory mediators, causing multi-organ dysfunction, failure, and death (Kleinpell et al., 2006; Singer et al., 2016). Recently, the ECS has emerged as a potential therapeutic target in sepsis treatment due to its immune modulatory functions. The effect of modulation of CB_1_R, CB_2_R, and GPR55 in sepsis-induced systemic inflammation will be described below.

### CB_1_R

Cannabinoid receptors type 1 are mainly pre-synaptic receptors in axon terminals and their activation causes post-synaptic inhibition by preventing neurotransmitter release (Chevaleyre et al., 2006; Ladak et al., 2011). Manipulating the activity of CB_1_R at critical periods may play a therapeutic role in sepsis. Studies have suggested that pre-synaptic CB_1_R activation on autonomic nerves and vascular walls exacerbates the hypotension associated with septic shock through neurogenic mechanisms (Godlewski et al., 2004). However, studies using CB_1_R knockout mice subjected to endotoxemia showed acute hypotension indicating that other mechanisms are also responsible for hypotension during systemic inflammatory conditions (Bátkai et al., 2004). In a lipopolysaccharide (LPS) induced experimental sepsis model, we demonstrated that inhibition of CB_1_R by its antagonist, AM281, significantly reduced leukocyte activation and improved intestinal microcirculation (Kianian et al., 2014) and iris microcirculation (Kelly et al., 2010; Al-Banna et al., 2013). However, the exact mechanisms of CB_1_R action in sepsis and septic shock are not yet completely understood and further studies are still required.

### CB_2_R

Cannabinoid receptors type 2 are primarily expressed on immune cells and represent an ideal target for immune modulation (Klein, 2005). CB_2_R are G_i_-protein coupled receptors and signal primarily through regulating cAMP levels depending on the duration of activation of the receptor (Rinaldi-Carmona et al., 1998; Börner et al., 2009; Basu and Dittel, 2011). CB_2_R also signal through the mitogen-activated protein kinase (MAPK) pathway by regulating the three major kinases: the extracellular signal-regulated protein kinases (ERK), p. 38, and c-Jun NH2-terminal kinases (Basu and Dittel, 2011). Both of these major signaling pathways play important roles in CB_2_R-mediated immune modulating functions including effects on leukocyte activation, migration, proliferation, apoptosis, and cytokine production (Basu and Dittel, 2011). In general, CB_2_R activation has shown an immune suppressive action, which can be exploited for therapeutic benefit in inflammatory diseases such as sepsis.

A variety of *in vitro* studies have shown contradictory results in terms of modulation of the immune response, mainly due to the use of non-selective cannabinoids (Miller and Stella, 2008). Some studies have shown that cannabinoids enhanced leukocyte proliferation in a dose dependent manner (Derocq et al., 1995; Carrier et al., 2004), while other studies have shown inhibitory effects on leukocyte proliferation through the activation of the CB_2_R (Maresz et al., 2007; Basu and Dittel, 2011). This inhibition maybe mediated by CB_2_R-dependent promotion of apoptosis in dendritic cells, splenocytes, and thymocytes, with some diminished activity when CB_2_R antagonists are used (Basu and Dittel, 2011). Additional evidence also supports the role of CB_2_R in the promotion of apoptosis. For example, administration of the CB_2_R agonist, JWH-015, induced apoptosis in thymocytes and diminished the proliferative potential of T cells and B cells (Lombard et al., 2007). Administration of the CB_2_R antagonist, AM630, showed a reversal of the induction of T cell apoptosis by JWH-133 (another CB_2_R agonist), strongly implicating a CB_2_R dependent mechanism (Singh et al., 2012). This evidence suggests that activation of CB_2_R may promote immune resolution by inducing apoptosis of immune cells, therefore minimizing excessive damage of the pro-inflammatory cascade that occurs early on in sepsis.

Using an experimental sepsis model, we demonstrated that activation of CB_2_R by the selective CB_2_R agonist, HU308, significantly reduced leukocyte adhesion in the microvasculature (Lehmann et al., 2012). Administration of EC degradation enzyme inhibitors, such as URB597 and JZL184, also decreased leukocyte activation in endotoxemic animals (Sardinha et al., 2014). However, reduced leukocyte activation by JZL184 is still present in endotoxemic CB_2_R knockout mice, suggesting that other mechanisms are also involved in the ECS-mediated immune regulation in sepsis.

Cytokine production by immune cells plays a critical role in the inflammatory response and can be modulated through CB_2_R. Multiple pro-inflammatory cytokines, such as TNF-α, interleukin (IL)-1β, and IL-6, are released in the early stages of the septic cascade. However, activation of the CB_2_R by its agonist HU308 reduced plasma levels of pro-inflammatory cytokines in endotoxemic rats (Lehmann et al., 2012). Administration of the EC, anandamide, decreased the levels of the proinflammatory cytokines IL-12 and IL-23 *in vitro* in activated microglial cells (Correa et al., 2009). In addition, T cell activation and release of IL-2 were inhibited by administration of the CB_2_R agonist, JWH-015, and this effect was eliminated by administration of the CB_2_R antagonist, AM630 (Börner et al., 2009). It was also demonstrated that activation of CB_2_R by HU308 enhanced the release of IL-10, a prominent anti-inflammatory cytokine, suggesting an immunosuppressive effect of CB_2_R (Klein, 2005).

### GPR55

GPR55 was initially described as a novel cannabinoid receptor or putative “CB_3_” receptor due to its high affinity to cannabinoid ligands such as Δ9-THC, 2-AG, anandamide, and rimonabant, independent of the presence of CB_1_R and CB_2_R (Sawzdargo et al., 1999; Begg et al., 2005; Pertwee, 2007; Ryberg et al., 2007). However, the limited sequence similarity between GPR55 and CBR does not support this concept (Baker et al., 2006). Unlike the classical CB_1_R and CB_2_R signaling pathway, GPR55 is coupled to Gα12 and Gα13 proteins, signaling through ras homolog gene family member A, Rho-associated protein kinase and phospholipase C pathway activation. Increased intracellular Ca^2+^ is followed to activate rhoA, Rac, and cdc42, thereby phosphorylating ERK, resulting in modulation of leukocyte chemotaxis, proliferation, and cytokine production (Ryberg et al., 2007; Henstridge et al., 2009).

GPR55 is widely expressed in the CNS, immune system, and peripheral tissues and is involved in many physiological and pathophysiological processes (Ryberg et al., 2007; Henstridge et al., 2011). In the immune system, GPR55 is highly expressed in the spleen and leukocytes, and its role in the modulation of innate and adaptive immune responses suggests a potential therapeutic effect for sepsis (Staton et al., 2008; Lin et al., 2011; Schicho and Storr, 2012; Stančić et al., 2015). GPR55 acts as an essential regulator in innate immunity via stimulatory effects in neutrophils, mast cells, monocytes, and natural killer (NK) cells (Balenga et al., 2011; Cantarella et al., 2011; Schicho et al., 2011; Chiurchiù et al., 2015). GPR55 on NK cells and monocytes increase pro-inflammatory cytokines, cell cytotoxicity, and decrease monocyte-mediated endocytosis upon activation by LPS (Chiurchiù et al., 2015). GPR55 expression was increased in the GI tract during sepsis (Lin et al., 2011) and GPR55 knockout mice showed least severe intestinal inflammation in comparison to CB_1_R or CB_2_R knockout mice in experimental colitis (Schicho and Storr, 2012). In studies using adjuvant-induced inflammation, inflammatory mechanical hyperalgesia by Freund’s complete adjuvant was absent in GPR55 knockout mice with increased levels of IL-4, IL-10, and IFN-γ (Staton et al., 2008). Importantly, GPR55 antagonist, CID16020046, diminished inflammation in experimental colitis by reducing the levels of pro-inflammatory cytokines, TNF-α, IL-1β, IL-6, and impairing leukocyte activation and migration (Stančić et al., 2015). In our laboratory, we demonstrated that GPR55 antagonists, CID16020046, and O-1918, reduced LPS-induced leukocyte-endothelial interactions in experimental models of sepsis in mice (Yang et al., 2016b).

GPR55 pharmacology with regards to ligand affinity and activity has been controversial in the current literature due to ligand- and concentration-specific biased signaling (Henstridge et al., 2010; Zeng et al., 2015). GPR55 can form heteromers with CB_1_R or CB_2_R to elicit different pathways via ligand- and concentration-specific crosstalk (Balenga et al., 2011, 2014; Kargl et al., 2012; Martínez-Pinilla et al., 2014). Heteromers of CB_1_R and GPR55 are reported in CNS (Martínez-Pinilla et al., 2014) and Human Embryonic Kidney cell lines (Kargl et al., 2012). CB_1_R inhibits GPR55 signaling when they are co-expressed on a cell (Kargl et al., 2012). Cross-interaction between GPR55 and CB_2_R modulates partner receptor mediated signaling. Co-expression of CB_2_R with GPR55 reduces GPR55 agonist-mediated activation of transcription factors, whereas CB2 receptor-mediated signaling was inhibited by co-expression with GPR55 (Balenga et al., 2014). GPR55-CB_2_R crosstalk in neutrophils was demonstrated by the finding that GPR55 activation led to augmented neutrophil chemotaxis and reduced CB_2_R-mediated tissue injury in the site of inflammation, suggesting a possible cellular mechanism of GPR55-mediated immune cell modulation (Balenga et al., 2011). Consequently, further investigations on ligand-specific signaling pathways are required to develop a specific pharmacological target for precise and designated immune modulation.

### ECS in CNS Injury

Central nervous system injury includes traumatic brain injury, stroke, cerebral aneurysms, and spinal cord injuries. Survivors from acute CNS injury often have complications due to infections. The incidence of fatal infections is linked to severity of CNS injury and the status of immune system (Klehmet et al., 2009; Shim and Wong, 2016). Following acute CNS injury, cell death occurs at the primary site and cytotoxins are released, which trigger significant secondary cell death outside the original injury area. In addition, function of the blood brain barrier is impaired, allowing systemic inflammatory mediators and cells to enter the normally protected CNS tissue, leading to the pathology of a CNS injury, i.e., neuroinflammation. The level of neuroinflammation is highly dependent on the severity, duration, and the anatomical context of the CNS injury. To prevent the excessive action of pro-inflammatory cytokines after their initial beneficial effects, the immune system releases several anti-inflammatory mediators, such as IL-10 and IL-1 receptor antagonist and soluble tumor necrosis factor receptors. This begins a cascade of compensatory anti-inflammatory response. Onset of an acute CNS injury also activates immunoinhibitory pathways, leading to a systemic brain-mediated immunosuppression to minimize secondary damage to healthy CNS tissue (Meisel et al., 2005; Haeusler et al., 2012). Systemic immunosuppression is believed to be the main reason for infections, a leading cause of death in patients with acute CNS injury. This increased susceptibility to infections, due to impaired immune function after an acute CNS injury, has been termed “CNS injury-induced immunodeficiency syndrome” (CIDS; Meisel et al., 2005).

Since the discovery of the ECS, its effects on the brain have prompted queries into its potential physiological and pathological roles. Local ECS is activated following CNS injury, representing an adaptive mechanism. The primary ligands produced in the brain are anandamide and 2-AG, which work on both CB_1_R and CB_2_R. This may play a role in modulation of CNS activity and regulation of the immune response after a CNS injury (Mechoulam et al., 1995; Sugiura et al., 1995; Bisogno et al., 1999). There are two different directions for the potential therapeutic use of CBR: neuroprotection and immunomodulation to reduce the CNS damage and improve the outcome.

One of the potential ways the brain protects itself is by reducing its excitatory activity. It is proposed that activation of presynaptic CB_1_R reduces the release of major excitatory neurotransmitters, such as glutamate (Coomber et al., 2008), which might be one of the earliest neuroprotective mechanisms deployed by the brain to prevent excitotoxicity. This is further supported by studies that showed blocking CB_1_R activity increased the vulnerability of neurons to ischemic damage and disrupted neuronal maintenance (Schweitzer, 2000; Hwang et al., 2010). Additionally, complete removal of the CB_1_R and associated signaling pathways causes an increase in susceptibility to ischemic damage, excitotoxin exposure, traumatic brain injury, and exacerbated inflammatory damage (Hillard, 2008). However, early inhibition of CB_1_R activation together with increased CB_2_R activation produces beneficial effects, such as a reduction of immune cells in cerebral vasculature, a reduction in infarct size, and an improved motor function after transient focal ischemia (Nagayama et al., 1999; Gilbert et al., 2007; Zhang et al., 2007; Adhikary et al., 2011).

The mechanism that may be related to the neuroprotective aspect of CB signaling is related to ERK in response to tissue insults and involved in cell survival mechanisms (Scotter et al., 2010). ERK activation is coupled to the presence of CB_1_R in hippocampal regions (Marsicano et al., 2003), suggesting that CB signaling is part of the compensatory response to CNS injury. Neuronal maintenance aspects of CB signaling seem to involve MAPK. Experimental support for this notion comes from studies that showed treating the hippocampal tissue with CB_1_R antagonist AM281 blocked ERK activation through MAPK kinase and led to a compromised neuronal survival (Karanian et al., 2005a). On the other hand, activating the CB_1_R and promoting cell survival also showed the neuroprotective action of the ECS through ERK activity (Jiang et al., 2005).

Cannabidiol, a main non-psychoactive component of cannabis, is suggested to exhibit some of its neuroprotective properties via inhibition of EC deactivation or even through its effects on vanilloid and 5-HT receptors (Mishima et al., 2005; Alvarez et al., 2008; Pazos et al., 2013). Other studies have shown that anandamide, 2-AG, THC, and synthetic agonists of CB_1_R also exhibit similar neuroprotective effects (Nagayama et al., 1999; Panikashvili et al., 2001). Endogenous anandamide showed neuroprotective properties in the developing brain through CB_1_R activity (van der Stelt et al., 2001; Shouman et al., 2006). Administration of 2-AG to animals with CNS injury reduced brain edema, infarct volume, and hippocampal cell death, and improved behavioral scores, suggesting better recovery (Shohami et al., 1997; Panikashvili et al., 2006). The excitotoxic protective property of ECS activation has been reversed by administration of CB_1_R and CB_2_R antagonists, AM281 and AM630, respectively. Other CB_1_R antagonists, such as SR-141716A have been shown to reduce or completely abolish the neuroprotective properties of EC signaling in transient global cerebral ischemia (Nagayama et al., 1999; Marsicano et al., 2002). Despite piling evidence suggesting the neuroprotective role of ECS, inconsistent results and outcomes are produced. The inconsistency is due to various factors, such as the delicacy of the physiological conditions, their severity, the timing of the pathologic development of a CNS injury and the pharmacological intervention. Therefore, careful consideration needs to be given to pharmacological modulation of ECS via CBR in terms of dosage and timing of administration, otherwise the results may be counterproductive or even harmful.

Enhancing EC actions by targeting its degradation represents an alternative therapeutic approach and has shown promising results in neuroprotection. The enzyme fatty acid amide hydrolase (FAAH) is responsible for anandamide breakdown. Pharmacologic inhibition or genetic knockout of FAAH promotes neuronal maintenance and function (Hwang et al., 2010; Celorrio et al., 2016). Block of anandamide transport with AM404 promotes CB_1_R signaling and enhances protection against excitotoxicity in hippocampal slices (Karanian et al., 2005b). Moreover, monoacylglycerol lipase (MAGL) hydrolyzes 2-AG to generate a major arachidonate precursor pool for neuroinflammatory prostaglandins, and is suggested as a potential drug target in neurodegenerative disease (Nomura et al., 2011; Fernández-Suárez et al., 2014). Although there is no direct evidence suggesting the benefit of MAGL inhibition after CNS injury, we can speculate that the involved pathways could be targeted to suppress proinflammatory cascades, which arise after an acute CNS injury and contribute to exacerbated CNS damage.

Due to the changes in the immune status after an acute CNS injury and the onset of CIDS, CB_2_R expression profile on immune cells and other non-neuronal cells suggested a potential theoretical association between the detrimental effects of CNS injury and CB_2_R activity. The immune impairment could potentially be modulated through the activity of the CB_2_R, ultimately making the patient susceptible to common infections and worsening the outcome. In general, CB_2_R agonists attenuate the inflammatory response by inhibiting production of pro-inflammatory mediators, decreasing immune cell chemotaxis and reducing extravasation in the vulnerable CNS (Shohami et al., 2011; Sardinha et al., 2014). Multiple studies have shown CB_2_R activation to be associated with neuroprotection and even improved blood brain barrier function (Ramirez et al., 2012). While many studies have established that the CB_2_R activation initiates immunosuppressive mechanisms and potentially limits neuroinflammation (Benito et al., 2008), others have suggested that the time-course of CB_2_R activity may hold the solution by avoiding the negative effects of neuroprotective immunosuppression, while still receiving the neuroprotective aspects of reduced neuroinflammation (Lehmann et al., 2014). Specifically, it is suggested that the inhibition of CB_2_R that is done too early could potentially increase the size of CNS injury, as the proinflammatory cascades and neutrophil infiltration will continue to develop. In our laboratory, we have demonstrated inhibition of CB_2_R by the selective antagonist, AM 630, significantly increased immune function as indicated by an increased leukocyte adherence to endothelia in animals challenged with LPS after hypoxia-ischemia (HI)-induced CNS injury. The CB_2_R inhibition did not affect the magnitude of infarct size in the injured brain (Burkovskiy et al., 2016). This outlines both the complexity of the CNS injury pathology, as well as the associated ECS signaling pathways, which remain to be fully explored.

## Conclusion

Although it has been shown that the ECS plays a vital role in the function of the immune system, controversial results exist for its regulatory role in sepsis, mainly due to the variety of methods employed to activate the receptors and the lack of truly selective ligands. In addition, *in vivo* studies using CB_2_R knockout mice showed conflicting results, which might be attributed to the complexity of the inflammatory models used in mimicking a septic state. There is a growing body of evidence for a pro-inflammatory role of GPR55 in sepsis, suggesting that selective GPR55 antagonists have a potential as a modulators of the immune response, and can be designed as a therapeutic target in sepsis.

With regards to the role of the ECS following CNS injury one may feel that cannabinoid signaling entails the “magic bullet” approach to many of the detrimental impairments associated with CNS injury. However, not all aspects of cannabinoid signaling have been fully explored and extensive pre-clinical testing is essential to find the correct ligand (or combination of ligands). Moreover, many of the studies have demonstrated a close association between ECS activity and improved functional outcome, reduced neurotoxicity, reduced infarct volume, and other beneficial effects. While this definitely suggests a bright future for this field, potential detrimental effects of ECS modulation need to be studied in more detail to prevent unwanted side effects.

## Author Contributions

Each author has contributed significantly to this work by writing a section of the manuscript, including its references. JZ: Introduction; Integrating and drafting the manuscript, revising and final approval of the version; IB: ECS in CNS injury; HY: GPR55 of ECS in sepsis; JS: ECS in sepsis, CBR1 and CBR2; CL: Conclusion; revising and final approval.

## Conflict of Interest Statement

The authors declare that the research was conducted in the absence of any commercial or financial relationships that could be construed as a potential conflict of interest.
